# Integration of single-cell and RNA-seq analysis reveals sepsis heterogeneity and prognostic significance of FCGR3A+ Macrophage subtypes

**DOI:** 10.1016/j.bbrep.2025.102352

**Published:** 2025-11-19

**Authors:** Yijia He, Shuanghu Dong, Lifen Ouyang, Jun Kang, Liying Chen, Qingyun He

**Affiliations:** a Department of Intensive Care Unit, Jiangxi Province Hospital of Integrated Traditional Chinese and Western Medicine, 330003, China; b The First Clinical Medical College, Gannan Medical University, Ganzhou, 341000, China; c Affiliated Hospital of Jiangxi University of Chinese Medicine, Nanchang, Jiangxi, 330006, China

**Keywords:** FCGR3A, Sepsis, Single cell and RNA-Seq, Immunology, Macrophage subtypes

## Abstract

Sepsis, a pressing global health challenge, is characterized by immune dysregulation and high mortality. This study, in response to this urgent need, integrated Single-cell RNA sequencing data from 53 samples and bulk transcriptomic data from 479 sepsis patients to analyze immune microenvironment heterogeneity and develop a prognostic signature. Using the scVI algorithm, eight immune cell types were identified. B, T, and NK cells were reduced in sepsis, while neutrophils were elevated. “BayesPrism” analysis linked higher macrophage abundance to improved survival. A pro-inflammatory FCGR3A + macrophage (Macro_1_FCGR3A) subset exhibited high M1 scores, enhanced antigen presentation, and activation of the PI3K-AKT-mTOR and TNF-NFκB pathways. Pseudotime analysis showed terminal differentiation with elevated HLA-DRB1 and CXCL10. A prognostic model using “StepCox + Ridge” selected 13 key genes, including CX3CR1, achieving AUCs (Area Under the Curve) of 0.77 (training) and 0.69 (validation). CX3CR1 showed strong diagnostic potential (AUC = 0.982). These findings reveal the pivotal role of FCGR3A + macrophages in sepsis prognosis.

## Highlights

• Single-Cell Sequencing and Cell Type Annotation Reveal Heterogeneity in the Immune Microenvironment of Sepsis.
• FCGR3A + macrophage subset and sepsis survival.
• Machine learning-based prognostic feature construction.

## 1 Introduction

Sepsis is a major global health threat, with its high mortality closely associated with long-term organ dysfunction in survivors [1]. According to the Sepsis-3 definition, sepsis is defined as organ dysfunction caused by infection, characterized by immune response dysregulation induced by infection that leads to multi-organ failure [2]. Recent epidemiological studies indicate that in 2017, there were approximately 48.9 million cases of sepsis globally, with 11 million deaths attributed to organ dysfunctions [3]. Given its significant public health burden, sepsis was declared a priority global health disease by the World Health Organization (WHO) in 2020 [4]. Despite increased attention on sepsis, current research regarding treatments has not yielded encouraging results [5]. While early fluid resuscitation and antibiotic therapy have improved outcomes for some patients, the "immune suppression - excessive inflammation" dual-hit caused by immune dysregulation continues to lead to treatment failure [6]. The primary reason for these outcomes is the significant heterogeneity among sepsis patients; thus, in-depth exploration of sepsis pathogenesis and pathological changes is critical to addressing the current treatment challenges.

Traditional diagnostic methods based on blood biomarkers, such as procalcitonin and C-reactive protein, are limited in reflecting the dynamic changes in immune cells [7,8], and no biomarker currently exists to definitively diagnose sepsis or predict clinical outcomes, leading to a poor prognosis in patients with sepsis [9]. Breakthroughs in single-cell sequencing technology have provided a new perspective for unraveling the immune microenvironment heterogeneity in sepsis. Several single-cell sequencing-based studies have revealed significant abnormalities in immune cell subset differentiation in sepsis patients. Macrophages, as core executors of innate immunity, play a critical role in disease progression through the imbalance of their functional polarization (M1/M2) [10]. In our analysis, a distinct macrophage subset, labeled as Macro_1_FCGR3A, was identified, exhibiting a unique pro-inflammatory profile. This subset demonstrated high M1 scores, enhanced antigen presentation, and activation of key immune signaling pathways, including PI3K-AKT-mTOR and TNF-NFκB. As a key receptor for immune regulation, FCGR3A is involved in both antibody-dependent cellular cytotoxicity and in modulating macrophage activation, further contributing to immune dysregulation in sepsis. Additionally, studies by Ren-Qi Yao et al. have identified specific cell subgroups, such as S100A9+ monocytes, which are associated with the immune-suppressive state during septic insult. Suppression of S100A9+ monocytes can hugely alleviate sepsis-induced immune suppression [11]. Despite progress in understanding the pathological mechanisms of sepsis, the role of immune cell heterogeneity and its dynamic changes during disease progression remains unclear. Traditional bulk transcriptomic analysis struggles to identify immune microenvironment differences at the single-cell level. In contrast, single-cell sequencing technology provides a new view for exploring immune cell subset differentiation, functional status, and intercellular communication in sepsis [[12], [13], [14]]. Therefore, there is a need to integrate large-scale single-cell datasets to explore the pathological mechanisms of sepsis.

This study integrates multi-omics data, including single-cell sequencing and bulk transcriptomic data from sepsis patients, to systematically analyze the immune cell composition characteristics in sepsis. We focus on exploring the heterogeneity of macrophages, neutrophils, and T/NK cell subgroups and their association with patient prognosis. Additionally, we construct a machine learning prediction model based on single-cell features, aiming to provide new therapeutic targets for precision sepsis diagnosis and new tools for personalized survival prediction of sepsis patients.

## 2 Materials and methods

### 2.1 Sample Collection

In this study, three publicly available single-cell sequencing datasets on sepsis were obtained from the GEO database: GSE175453 [15], GSE167363 [16], and GSE216009 [17]. A total of 53 single-cell sequencing samples were included, comprising 13 standard whole blood samples and 40 sepsis samples. Additionally, a bulk transcriptomic gene expression profile and clinical data were collected from the GSE65682 dataset [18], which included 760 sepsis patients and 42 healthy individuals. Of these, 479 samples had complete survival data.

### 2.2 Single-cell sequencing analysis

Single-cell analysis was performed using the “scanpy” Python package [19] (18). Doublets were filtered out using “scrublet” [20], and cells expressing multiple cell-type markers were removed. Additional filters excluded cells with feature counts exceeding 5000, gene expression counts below 300, mitochondrial gene expression percentages above 8 %, and erythrocyte gene expression percentages above 1 %. High-quality cell expression data were identified, and the top 2000 highly variable genes were selected. The “scVI” package [21] was then used for batch correction across datasets with the following parameters: n_layers = 2, n_latent = 30, and gene_likelihood = "nb". After integration, unsupervised clustering (resolution = 1) and UMAP (Uniform Manifold Approximation and Projection) dimensionality reduction were applied (parameters: min_dist = 0.3). The Leiden method was used for unsupervised clustering with default parameters (n_pcs = 30, n_neighbors = 20). Each cluster was annotated using known cell markers: macrophages (*CD86, CD68, CD163*), neutrophils (*CSF3R*, S100A8*, FCGR3B*), T cells (*CD3D, CD3E, CD3G, CCR7, CD8A, CD8B, CD4, FOXP3, IL2RA*), NK cells (*NKG7, GNLY, KLRF1*), B cells (*MS4A1, CD79A, CD19, CD79B*), plasma cells (*IGHG1, JCHAIN*), and platelets (*PPBP*) (16).

### 2.3 Bayesian prism deconvolution

The BayesPrism algorithm was used to deconvolute macrophages, T cells, NK cells, neutrophils, B cells, plasma cells, and platelets [22]. This approach was used on cell states, cell types, gene expression, and bulk RNA-seq data to estimate the proportions of individual cell states. Analyses utilized TPM-normalized data, visualizing pairwise correlations between cell types, outlier gene distributions, and running BayesPrism with default parameters.

### 2.4 Pseudotime trajectory analysis of macrophages

The “cytotrace2” R package [23] was employed to predict the differentiation potential of macrophages, which served as the starting point for pseudotime analysis. The “monocle3” package was used to construct pseudotime trajectories, identifying developmental paths and transitions among macrophage subpopulations [24,25].

### 2.5 Cell communication analysis

The “CellChat2” R package [26] was used to construct cellular communication networks that are based on receptor-ligand interactions. The analysis identified communication probabilities and visualized interactions and network strengths between different cell groups.

### 2.6 Functional enrichment analysis

Gene Ontology (GO) and Kyoto Encyclopedia of Genes and Genomes (KEGG) enrichment analyses were conducted using the “ClusterProfiler” R package [27,28] and “org.Hs.eg.db” for pathway enrichment. The “irGSEA” [29] package (https://github.com/chuiqin/irGSEA/) was used for gene set enrichment across different cell types, which incorporates multiple scoring methods and robust rank aggregation to identify consistently significant gene sets.

### 2.7 Machine learning model construction

The “Findallmarkers” function in the “Seurat” R package [30] identified markers for the FCGR3A + macrophage subtype. After a thorough data split into training (70 %) and validation (30 %) sets, univariate Cox regression was used to select prognostic genes. The “mime1” R package [31], which combines 101 machine learning methods, was meticulously employed to build the final sepsis prognosis model and select the optimal predictive model, ensuring the validity of our machine learning model.

### 2.8 Statistical analysis

All computations and analyses were conducted using Python and R. Student's t-test was used for comparing continuous variables between two groups. A two-sided p-value <0.05, a crucial threshold in statistical analysis, was considered statistically significant, underlining the importance of our findings.

## 3 Results

### 3.1 Single-cell sequencing and Cell Type Annotation Reveal Heterogeneity in the Immune Microenvironment of Sepsis

**Fig. 1:**
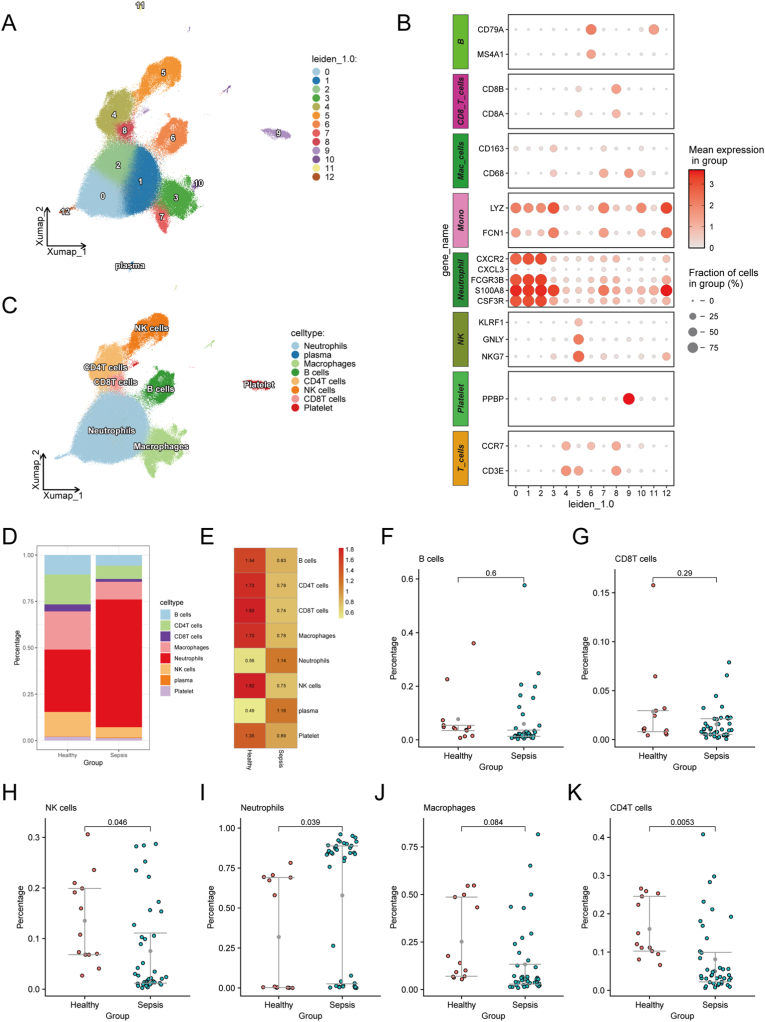
Single-cell sequencing and cell-type analysis reveal the heterogeneity of the immune microenvironment in sepsis. (A) UMAP plot showing the clustering of 220,656 single cells from 53 sepsis patients. Each point represents a cell, colored by cell type. (B) Dot plot of marker gene expression for each identified cell type. (C) Cell population annotation based on marker genes, including neutrophils, T cells, NK cells, B cells, macrophages, plasma cells, and platelets. (D) Proportions of immune cell types in sepsis patients vs. healthy controls. (E) Ro/e analysis showing preferential enrichment of B cells, macrophages, CD4^+^ T cells, CD8^+^ T cells, and NK cells in healthy controls. (F–H) Quantification of B cells, CD8^+^ T cells, and macrophages in healthy vs. sepsis patients. (I–J) Proportions of CD4^+^ T cells and NK cells in healthy vs. sepsis patients. (K) Neutrophil proportions were higher in sepsis patients.

To elucidate the cellular composition of sepsis, we utilized single-cell RNA sequencing (scRNA-seq) data from the GEO datasets GSE216009, GSE175453, and GSE167363, which collectively included peripheral blood samples from 53 sepsis patients. After stringent quality control, the data were integrated and batch effects were corrected using the "Scanpy" and "scVI" Python packages. A total of 220,656 high-quality cells were retained for downstream analysis. Subsequently, dimensionality reduction and clustering were performed using the "Scanpy" package, and UMAP visualization revealed 12 distinct cell clusters at a resolution of 1.0 (Fig. 1A). Cell populations were annotated based on canonical marker genes derived from published literature, as illustrated in the dot plot (Fig. 1B). Identified clusters included neutrophils, CD8T cells, CD4T cells, NK cells, B cells, macrophages, plasma cells, and platelets (Fig. 1C). Comparative analysis of immune cell proportions between sepsis patients and healthy controls demonstrated significant shifts: B cells, Macrophages, CD4^+^ T cells, CD8^+^ T cells, and NK cells were reduced in sepsis patients, whereas neutrophils were elevated (Fig. 1D). Single-cell Ro/e (Ratio of Observed over Expected cell number) analysis further revealed that B cells, macrophages, CD4T cells, CD8T cells, and NK cells were preferentially enriched in healthy controls (Fig. 1E). Further quantification confirmed that B cells, CD8T cells, and macrophages were higher in healthy individuals, though statistical significance was not reached (Fig. 1F–H). In contrast, CD4T cells and NK cells were copiously more abundant in healthy controls (Fig. 1I–J), while neutrophils dominated in sepsis patients (Fig. 1K).

### 3.2 Macrophage subset analysis

**Fig. 2:**
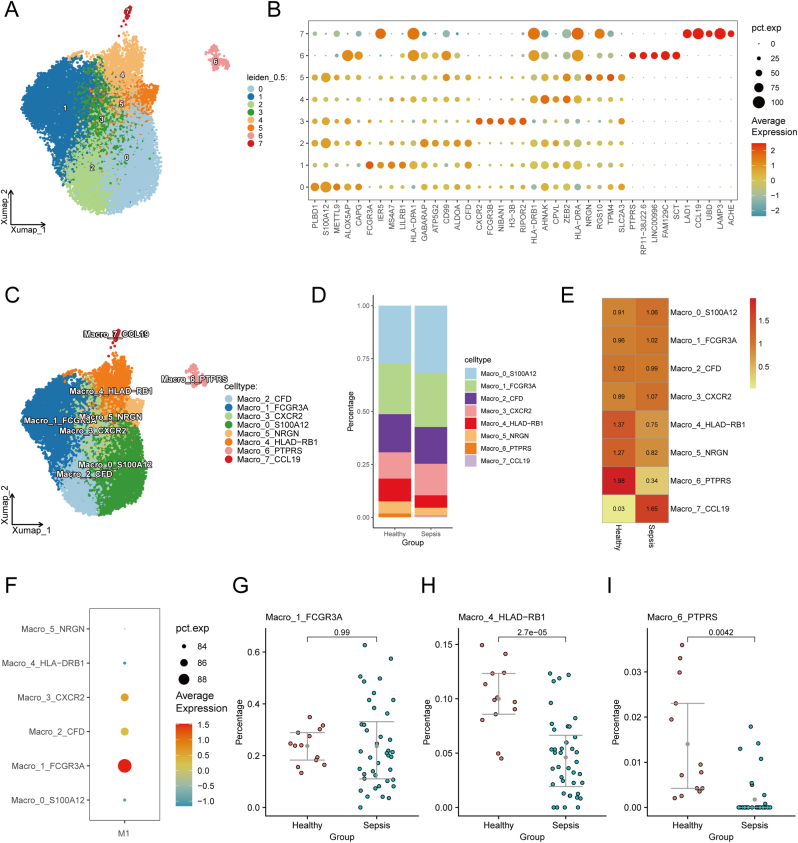
**Macrophage subset analysis.** (A) UMAP plot showing eight macrophage subsets identified at resolution = 0.5. (B) Marker genes for each macrophage subset. (C) Annotation of macrophage subsets: Macro_0_S100A12, Macro_1_FCGR3A, Macro_2_CFD, Macro_3_CXCR2, Macro_4_HLA-DRB1, Macro_5_NRGN, Macro_6_PTPRS, Macro_7_CCL19. (D) Proportions of macrophage subsets in healthy and sepsis patients. (E) Macro_4_HLA-DRB1, Macro_5_NRGN, and Macro_6_PTPRS were more abundant in healthy individuals. (F) Functional activity scores of macrophage subsets based on M1 macrophage genes. (G–I) Proportions of macrophage subsets in healthy vs. sepsis patients. Statistical significance determined by Student's t-test (p < 0.05).

Isolated macrophages were re-clustered with a resolution of 0.5, resulting in eight distinct subsets (Fig. 2A). Differential expression analysis (log2FC > 0.5, expression detected in at least 50 % of cells per cluster) identified marker genes for each cluster, with the top five shown in Fig. 2B. Based on these markers, clusters were annotated as Macro_0_S100A12, Macro_1_FCGR3A, Macro_2_CFD, Macro_3_CXCR2, Macro_4_HLA-DRB1, Macro_5_NRGN, Macro_6_PTPRS, and Macro_7_CCL19 (Fig. 2C). The proportions of these subsets in healthy and sepsis patients’ blood are shown in Fig. 2D. Macro_4_HLA-DRB1, Macro_5_NRGN, and Macro_6_PTPRS were preferentially found in healthy individuals (Fig. 2E). Using the AUCell package, we calculated functional activity scores for macrophage subsets based on M1 macrophage-related genes, finding that Macro_1_FCGR3A displayed the highest M1 score (Fig. 2F), suggesting it plays a central role in the sepsis immune response. Differential proportion analysis revealed that Macro_4_HLA-DRB1 and Macro_6_PTPRS were more abundant in healthy individuals, while Macro_1_FCGR3A showed no significant difference between the two groups (Fig. 2G–I).

### 3.3 Neutrophil subset analysis

**Fig. 3:**
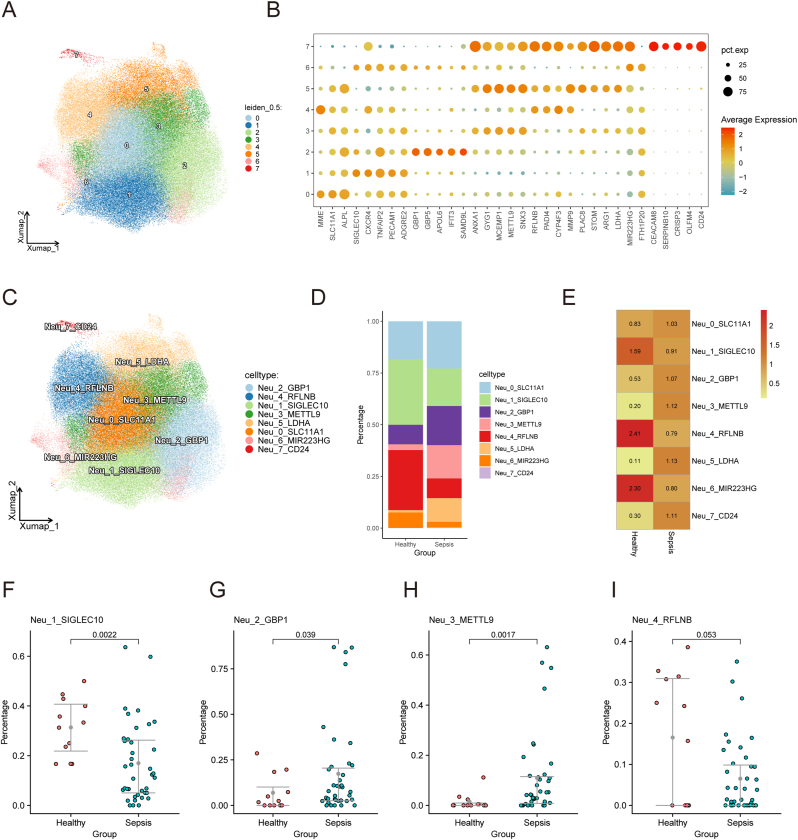
**Neutrophil subset analysis.** (A) UMAP plot showing eight neutrophil subsets. (B–C) Marker genes for each neutrophil subset. (D) Proportions of neutrophil subsets in healthy and sepsis patients. (E) Neu_1_SIGLEC10, Neu_4_RFLNB, and Neu_6_MIR223HG were more abundant in healthy controls. (F–I) Differential proportions between healthy and sepsis patients. p-values determined by Student's t-test.

Isolated neutrophils were re-clustered at a resolution of 0.5, resulting in eight subsets (Fig. 3A). Differential expression analysis identified marker genes for each cluster, which were annotated as Neu_0_SLC11A1, Neu_1_SIGLEC10, Neu_2_GBP1, Neu_3_METTL9, Neu_4_RFLNB, Neu_5_LDHA, Neu_6_MIR223HG, and Neu_7_CD24 (Fig. 3B and C). Neu_1_SIGLEC10, Neu_4_RFLNB, and Neu_6_MIR223HG were more prevalent in healthy individuals (Fig. 3D). Comparison of proportions showed that Neu_1_SIGLEC10 (p = 0.0022) and Neu_4_RFLNB (p = 0.053) were more abundant in healthy individuals, while Neu_2_GBP1 (p = 0.038) and Neu_3_METTL9 (p = 0.017) were more prevalent in sepsis patients (Fig. 3F–I).

### 3.4 T and NK cell subset analysis

**Fig. 4:**
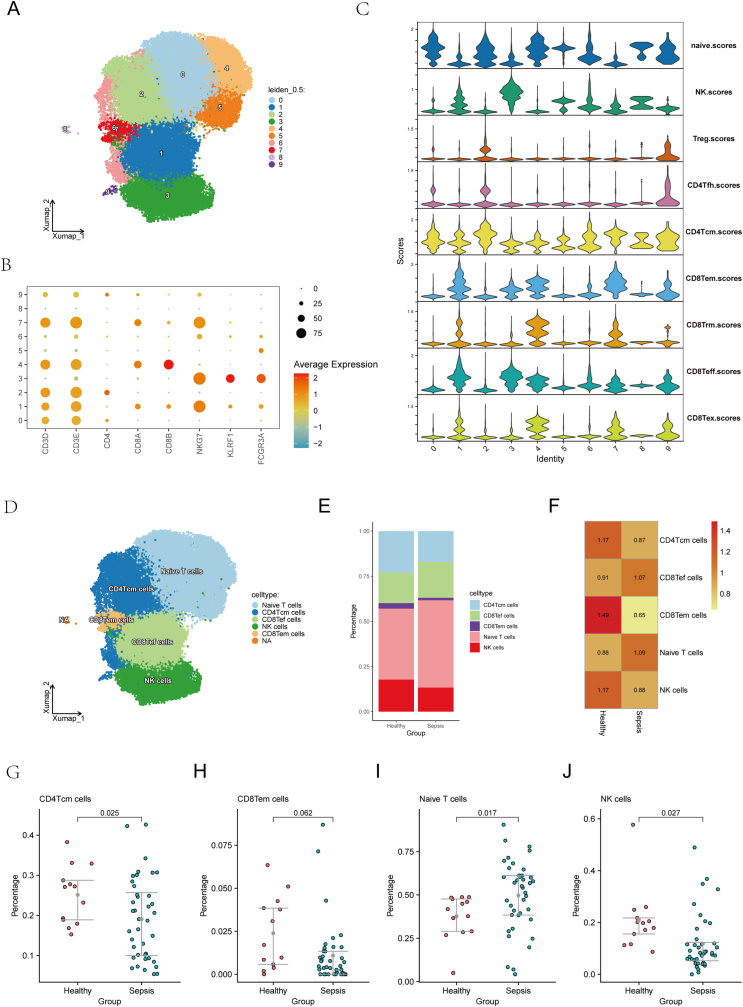
**T and NK cell subset analysis.** (A) UMAP plot of CD8^+^ T cells, CD4^+^ T cells, and NK cells at resolution = 0.5. (B) Canonical markers for T and NK cell subsets. (C) Functional activity scores for lymphocyte subsets. (D) Consolidated populations of naïve T cells, CD4Tcm, CD8Teff, NK cells, and CD8Tem cells. (E) Proportions of T and NK cell subsets in healthy and sepsis patients. (F) CD4Tcm, NK, and CD8Tem cells enriched in healthy controls. (G–J) Proportions of T and NK cells in healthy vs. sepsis patients, with statistical significance (p < 0.05).

We next isolated CD8^+^ T cells, CD4^+^ T cells, and NK cells for further clustering at a resolution of 0.5, resulting in ten clusters (Fig. 4A). Preliminary annotation using canonical markers—T cells: CD3D, CD3E, CD4, CD8A, CD8B; NK cells: NKG7, KLRF1, FCGR3A—is shown in Fig. 4B. We then compiled marker gene sets for nine lymphocyte subsets (naïve T cells, NK cells, Treg cells, CD4Tfh cells, CD4Tcm cells, CD8Tem cells, CD8Trm cells, CD8Teff cells, CD8Tex cells) and scored each cluster with the AUCell package (Fig. 4C). Based on these scores, we consolidated clusters into five populations: naïve T cells, CD4Tcm cells, CD8Teff cells, NK cells, and CD8Tem cells (Fig. 4D). Their proportions in healthy versus septic blood are plotted in Fig. 4E. Tissue‐preference analysis revealed that CD4Tcm cells, NK cells, and CD8Tem cells were enriched in healthy controls (Fig. 4F). In comparative analysis, CD4Tcm cells (p = 0.025), CD8Tem cells (p = 0.062), and NK cells (p = 0.027) were higher in healthy blood, whereas naive T cells (p = 0.017) were more abundant in sepsis (Fig. 4G–J).

### 3.5 Macrophage proportion associated with sepsis survival

**Fig. 5:**
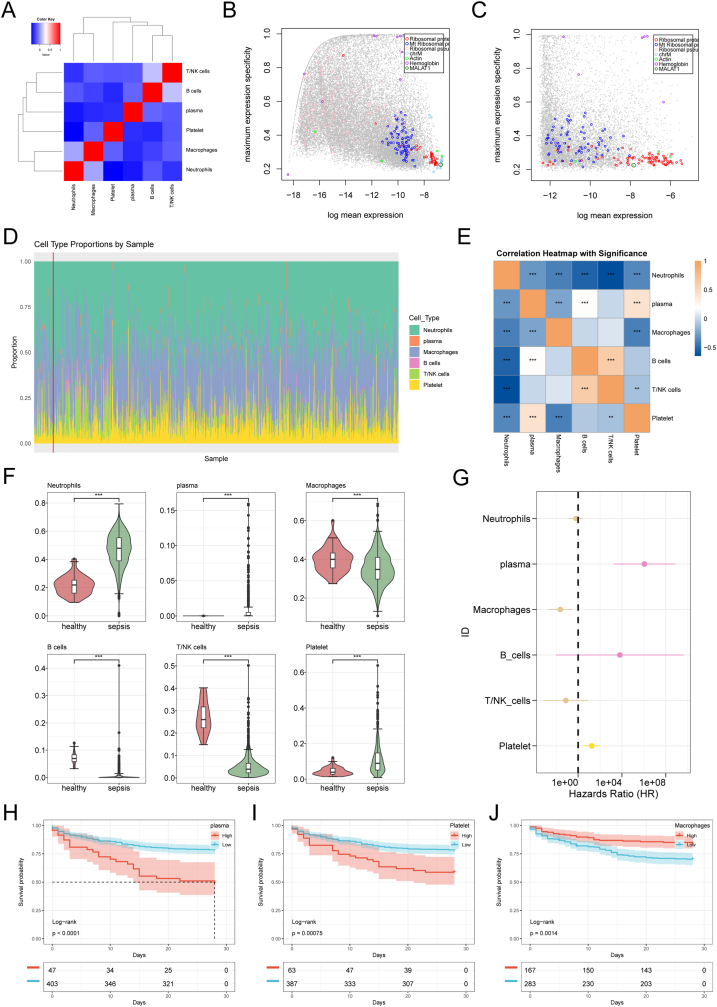
**Macrophage proportion associated with sepsis survival.** (A) Cell-type correlation matrix. (B) Outlier genes in the single-cell dataset. (C) Outlier genes in bulk RNA-seq data. (D) Proportion of cell types in bulk RNA-seq samples. (E) Pairwise correlations among cell types. (F) Comparison of cell-type proportions in healthy vs. sepsis patients. (G) Cox regression analysis showing high macrophage proportions as protective. (H–J) Kaplan-Meier survival analysis confirming macrophages as a protective factor.

To further explore the relationship between immune cell composition and survival in sepsis. Using our scRNA-seq data as a reference, we applied the BayesPrism algorithm to further explore the relationship between immune cell composition and survival in sepsis by inferring the proportions of each immune cell type within bulk RNA-seq profiles of septic blood samples. First, we constructed a cell-type correlation matrix by computing the average expression and specificity scores of each gene across all cell types (Fig. 5A). In the single-cell dataset, ribosomal and mitochondrial genes emerged as major outliers and were filtered out using the “cleanup.genes” function (Fig. 5B). Fig. 5C shows the corresponding outlier genes in the bulk-RNA-seq data. Subsequently, applying BayesPrism yielded the estimated proportions of each cell type in the bulk samples (Fig. 5D), with neutrophils and macrophages dominating most profiles. Next, we assessed pairwise correlations among cell types, finding that neutrophils negatively correlated with T/NK cells, B cells, macrophages, platelets, and plasma cells, whereas B cells were positively correlated with T/NK cells (Fig. 5E). We then compared the average cell-type proportions between healthy controls and septic patients: only neutrophil proportions were elevated in sepsis, while all other cell types (T/NK cells, B cells, macrophages, platelets, plasma cells) were reduced—mirroring our single-cell observations (Fig. 5F). Finally, to link cell proportions with outcome, we performed univariate Cox regression on each cell-type fraction; the resulting forest plot showed that a high macrophage fraction was protective, whereas high proportions of platelets and plasma cells were risk factors for sepsis patients (Fig. 5G). Kaplan–Meier analyses confirmed that patients with high macrophage proportions had more prolonged survival than those with low proportions, while those with high platelet or plasma-cell proportions had shorter survival compared to their low-proportion counterparts (Fig. 5H–J).

### 3.6 FCGR3A + macrophage subset and sepsis survival

**Fig. 6:**
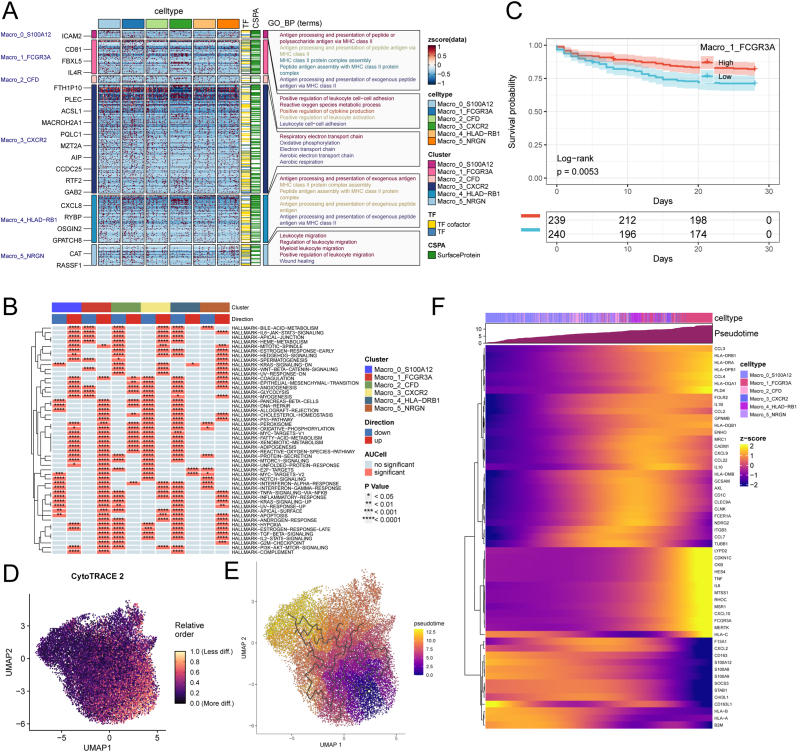
**FCGR3A + macrophage subset and sepsis survival.** (A) GO enrichment analysis of Macro_1_FCGR3A and Macro_4_HLA-DRB1 for antigen presentation pathways. (B) Hallmark pathway scoring using irGSEA showing upregulation of PI3K-AKT-mTOR and TNF-NFκB in Macro_1_FCGR3A. (C) GSVA-based survival analysis showing better survival for patients with a high Macro_1_FCGR3A signature. (D–E) Pseudotime analysis showing Macro_1_FCGR3A as the terminal state. (F) Gene expression in pseudotime showing increased immune activation and decreased M2 marker CD163.

To pinpoint which macrophage subset drives this effect, we focused on subtypes with >1000 cells and performed differential expression gene (DEG) analysis followed by GO enrichment. Macro_1_FCGR3A and Macro_4_HLA-DRB1 were enriched for antigen processing and presentation pathways via the MHC class II pathway, indicating roles in immune recognition and activation. Macro_3_CXCR2 enriched for mitochondrial respiratory and oxidative phosphorylation pathways (Fig. 6A). Using the “irGSEA” package, we scored hallmark pathways: Macro_1_FCGR3A showed downregulation of IL6-JAK-STAT3 but upregulation of PI3K-AKT-mTOR and TNF-NFκB signaling (Fig. 6B), suggesting an anti‐sepsis role. GSVA (gene set variation analysis) based scoring of each subtype's marker genes in bulk sepsis samples revealed that patients with a high Macro_1_FCGR3A signature had drastically better survival (Fig. 6C). Pseudotime analysis with “cytotrace2” and “Monocle3” showed Macro_0_S100A12 as the most multipotent and Macro_1_FCGR3A as the terminal state (Fig. 6D and E). Genes such as *TNF, CCL3, CCL4, CXCL9/10* and *HLA‐DRB1/DRA* increased along the trajectory, while M2 marker CD163 expression decreased (Fig. 6F), highlighting Macro_1_FCGR3A's role in immune activation and homeostasis.

### 3.7 Macro_1_FCGR3A and immune activation

**Fig. 7:**
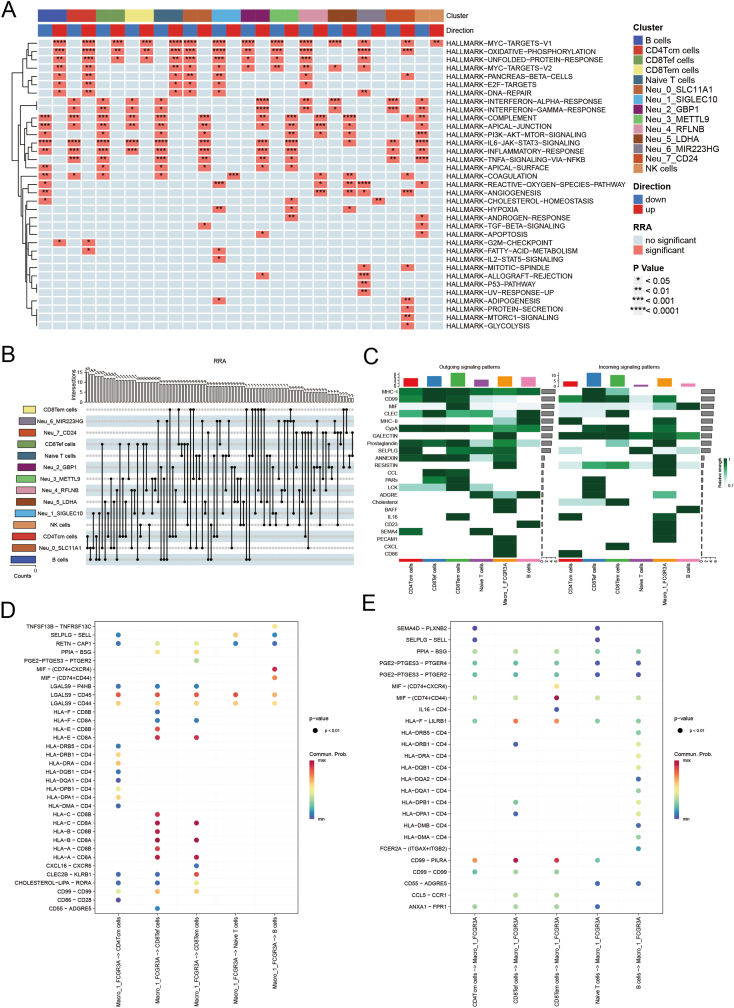
**Macro_1_FCGR3A and immune activation.** (A) irGSEA analysis of T cells, B cells, and NK cells showing reduced inflammatory-response signatures. (B) Upset plot showing pathway overlap among immune subsets. (C) CellChat ligand-receptor interaction map for Macro_1_FCGR3A. (D–E) Detailed ligand-receptor pairs, predominantly HLA interactions.

We applied irGSEA to T cells, B cells, and NK cells subsets, finding reduced inflammatory‐response and PI3K-AKT-mTOR signatures, implying functional impairment (Fig. 7A). An upset plot showed extensive pathway overlap among B, CD4Tcm cells, CD8Teff cells and CD8Tem cells (Fig. 7B). Using the “CellChat” package, we mapped ligand–receptor interactions: Macro_1_FCGR3A outputs MHC‐I, CYP, galectins, and SELPLG signals, and receives CD99, MHC‐II, CCL and IL16 signals (Fig. 7C). Detailed ligand–receptor pairs were predominantly HLA interactions (Fig. 7D and E), further confirming Macro_1_FCGR3A's pro‐inflammatory role.

### 3.8 Machine learning-based prognostic feature construction

**Fig. 8:**
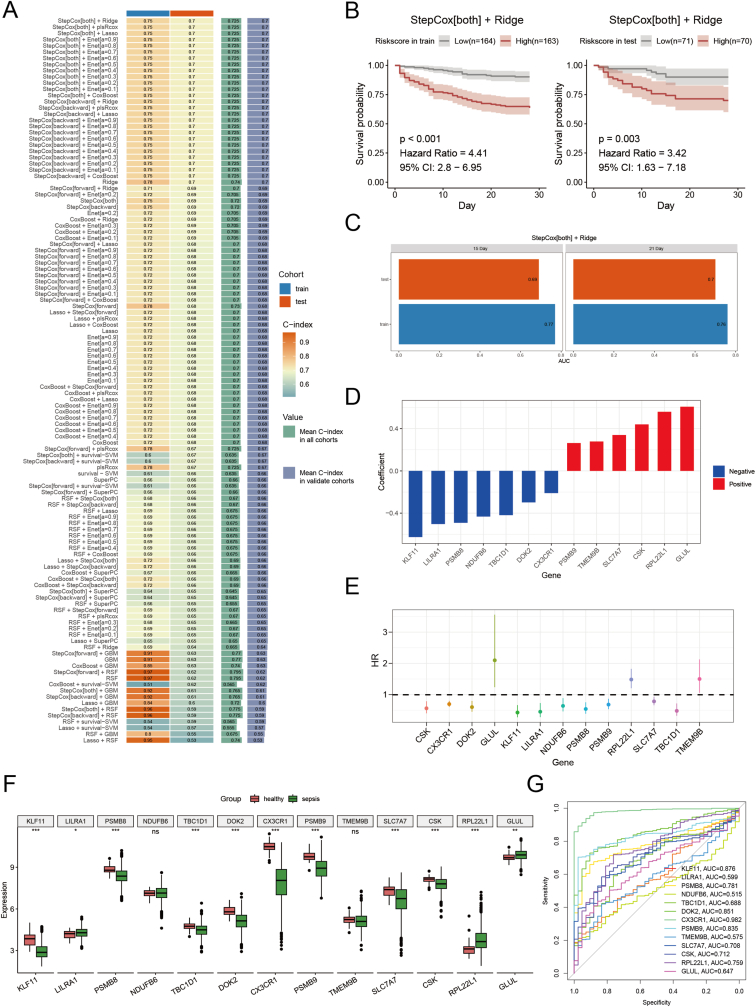
**Machine learning-based prognostic feature construction.** (A) C-index heatmap showing the best model ("StepCox[both] + Ridge") with mean C-index = 0.72. (B) Risk score analysis separating patients into high and low-risk groups. (C) AUC values at 15-day and 21-day survival points for the training and validation cohorts. (D) Regression coefficients for the 12 genes identified by Ridge regression. (E) Univariable Cox regression analysis of the model genes. (F) Boxplot showing the expression levels of the 12 genes in healthy and septic blood. (G) ROC curve analysis showing CX3CR1 as a promising biomarker (AUC = 0.982).

Given Macro_1_FCGR3A's survival association, we differentially expressed genes at logFC >0.2 in the subtype, yielding 746 genes, and intersected with bulk RNA‐seq to get 662. After filtering for complete survival data, 468 patients remained, split 70:30 into training and validation cohorts. We tested 101 ML pipelines (via the Mime1 package) and found the “StepCox[both] + Ridge” model—based on 13 genes—performed best (mean C‐index = 0.72; Fig. 8A). Risk scores separated patients: high‐risk patients survived shorter (HR = 4.41 training in the training cohort, HR = 3.42 in the validation cohort; Fig. 8B). AUCs at 15 and 21 days were 0.77 (15 days) and 0.76 (21 days) in the training cohort, and 0.69 (15 days) and 0.70 (21 days) in the validation cohort (Fig. 8C). Univariable Cox regression analysis confirmed most genes as protective except *GLUL, RPL22L1,* and *TMEM9B* (Fig. 8E). Expression analysis in healthy blood showed eight protective genes (e.g., *KLF11, PSMB8*) were more highly expressed, while the two risk genes (*RPL22L1, GLUL*) were lower (Fig. 8F), validating the model. ROC curve analysis highlighted four genes with AUC >0.8 (*CX3CR1* = 0.982), suggesting *CX3CR1* as a precise sepsis biomarker (Fig. 8G).

## 4 Discussion

This study systematically elucidated the heterogeneity of the immune microenvironment in sepsis patients by integrating single-cell sequencing with bulk transcriptomic data. For the first time, we identified the critical role of the FCGR3A + macrophage subset (Macro_1_FCGR3A) in disease prognosis, which is characterized by high expression of the FCGR3A and HLA-DPA1 genes. This subset was associated with immune maintenance and activation in sepsis patients. Our findings not only deepen the understanding of immune dysregulation mechanisms in sepsis but also provide new biomarkers and predictive tools that could revolutionize clinical diagnosis and treatment.

In this study, we integrated extensive single-cell datasets of sepsis and identified eight distinct cell types, including B cells, CD4T cells, CD8T cells, NK cells, Macrophages, Neutrophils, Platelets, and Plasma cells. The results confirmed a significant imbalance of immune cell proportions in sepsis patients. Specifically, the proportions of B cells, CD4T cells, CD8T cells, NK cells, and macrophages were reduced in sepsis patients, whereas neutrophils were notably elevated. This observation aligns with previous studies describing the biphasic imbalance of "immune exhaustion-excessive inflammation" in sepsis, where the disease is often characterized by T cell exhaustion, B cell depletion, and abnormal neutrophil activation, ultimately leading to host immune dysfunction [32]. Furthermore, our Ro/e analysis provided additional insights into the preferential distribution of different immune cells between healthy individuals and sepsis patients. We found that T cells, B cells, and NK cells were predominantly enriched in healthy individuals, whereas neutrophils were dominant in sepsis patients. These findings suggest that sepsis may lead to impairment of adaptive immunity, while excessive activation of innate immune cells could further exacerbate inflammatory damage.

Considering the impact of different immune cell subtypes on the survival of sepsis patients, we utilized the “BayesPrism” algorithm to quantify the proportion of immune cells in sepsis. The results revealed that macrophage proportion is significantly associated with sepsis patient survival. Macrophages are central drivers of immune dysregulation in sepsis, and their functional abnormalities have been a research focus over the past decade.

Focusing on macrophage heterogeneity, we identified a unique subset of macrophages with high expression of *FCGR3A*. The FCGR3A gene encodes the potent cytotoxic receptor CD16A, which is present on natural killer cells, monocytes, and macrophages and plays a crucial role in antibody-dependent cellular cytotoxicity [33]. Furthermore, we discovered that the Macro_1_FCGR3A subset exhibited a distinct pro-inflammatory phenotype. GO analysis indicated that this subset was mainly enriched in antigen processing and presentation-related pathway. Paradoxically, a higher proportion of this subset was associated with better patient prognosis.

This seemingly contradictory phenomenon may be attributed to its functional characteristics. Despite displaying M1-like polarization traits (high M1 scores), the subset also exhibits high expression of *HLA-DRB1* and *CXCL10*, which are involved in antigen presentation, as well as T cell-related cytokines and chemokines such as *TNF, CCL3, CCL4, CXCL9,* and *CXCL10*. These findings suggest that this subset may contribute to immune homeostasis by enhancing antigen presentation and promoting T-cell activation. Additionally, activation of the PI3K-AKT-mTOR pathway in the Macro_1_FCGR3A subset may have a “double-edged sword” effect; on one hand, it mitigates tissue damage by inhibiting excessive TLR4-NFκB activation [34](*33*); on the other hand, it sustains macrophage survival and functionality, which may be key to its protective prognostic effect.

This discovery aligns with the recently proposed "functional polarization continuum" theory, which suggests that the traditional M1-M2 dichotomy may be insufficient to describe the complex macrophage states in sepsis [10]. Notably, pseudotime trajectory analysis showed that this subset resides in the terminal differentiation stage, and its PI3K-AKT-mTOR pathway activation may help sustain its terminal differentiation state through metabolic reprogramming. This finding was consistent with macrophage metabolism studies, indicating that PI3K signaling enhances glycolytic flux to support a pro-inflammatory phenotype while simultaneously regulating lysosomal function via mTORC1 to maintain antigen-presenting capacity [35].

To validate the clinical significance of FCGR3A + macrophage-associated genes, we constructed a machine learning prediction model based on transcriptomic data from sepsis patients and identified 13 key genes. The machine learning model based on the characteristic genes of Macro_1_FCGR3A demonstrated stable performance in prognosis evaluation: the AUC values of the training set at 15 days and 21 days were 0.77 and 0.76, respectively, while the AUC values of the validation set at the same time points were 0.69 and 0.7, confirming the reliability of the model across different datasets. Further horizontal comparisons showed that the performance of this model is markedly superior to previous studies on sepsis prognosis based on immune-related genes and differentially expressed genes, highlighting its advantages in clinical prognosis evaluation [36,37]. Additionally, the model gene CX3CR1 demonstrated excellent diagnostic performance, with a diagnostic AUC value reaching 0.982. However, although CX3CR1 showed excellent diagnostic performance, its clinical application as a biomarker needs further validation to confirm its utility in sepsis diagnosis and prognosis.

Furthermore, gene expression analysis reinforced the prognostic value of the machine learning model, revealing that *CX3CR1, PSMB9*, and DOK2 were highly expressed in healthy individuals. At the same time, *GLUL* and *RPL22L1* were significantly upregulated in sepsis patients and correlated with poor prognosis. Notably, *CX3CR1* is a chemokine receptor involved in leukocyte adhesion and migration. Studies have demonstrated that *CX3CR1* deficiency induces macrophage polarization toward the M2 phenotype [38], whereas *CX3CR1* binding to its ligand *CX3CL1* promotes M1 polarization and regulates macrophage migration [39]. *PSMB9*, encoding the proteasome β subunit, is a critical component of the immunoproteasome, optimizing protein degradation to generate antigenic peptides suitable for MHC class I molecule presentation [40,41]. Additionally, a study by Ma Carmela P Dela Cruz demonstrated that *PSMB9* is highly expressed in surviving sepsis patients, supporting its accuracy as a prognostic biomarker [42].

The risk factor glutamine synthetase (*GLUL*) is an enzyme responsible for endogenous glutamine synthesis, playing a crucial role in metabolism and immune regulation. *GLUL* deficiency induces macrophage differentiation into a pro-inflammatory phenotype and exacerbates sepsis in mice [43]. Therefore, the high expression of GLUL in the Macro_1_FCGR3A subset may contribute to suppressing excessive inflammation and mitigating tissue damage. Although Macro_1_FCGR3A exhibits an M1-leaning phenotype with enhanced antigen presentation at the single-cell level and is substantially associated with improved survival, this putative protective association remains supported primarily by observational evidence. Drawing on our cell–cell communication and pathway-enrichment analyses, we hypothesize that this subset may mitigate immune dysregulation and reduce the risk of adverse outcomes by augmenting antigen presentation and promoting T-cell recruitment. Nevertheless, this hypothesis requires validation in controlled systems—using primary monocytes/macrophages and immune co-culture—through perturbation of key molecules (e.g., *CX3CR1, PSMB9, GLUL*) to establish causality.

Furthermore, compared with existing sepsis prognostic studies based on a single gene or small-scale gene sets [36,37,44], the innovations of this study lie in: (i) achieving cross-batch integration and unified annotation in multi-cohort single-cell data through “scVI”, thereby obtaining a robust immune cell atlas; (ii) extrapolating cell subpopulation differences at the single-cell level to large-scale bulk RNA-seq using “BayesPrism”, and quantifying the association between immune cell proportions and patient outcomes; (iii) focusing on the function and differentiation trajectory of Macro_1_FCGR3A, revealing their key role in antigen presentation and inflammatory pathways and their correlation with better survival; (iv) constructing and reporting transparent prognostic model parameters and performance evaluations based on the interpretable StepCox[both]+Ridge workflow, thus providing a basis for clinical transferability.

However, this study has several limitations: first, although batch correction was performed using the “scVI” algorithm, the heterogeneity of sample sources in public databases may affect the accuracy of cell proportion quantification. Second, the prognostic model lacks external validation with an independent cohort, requiring further confirmation through prospective clinical studies.

## Data availability statement

All authors agree that the data in the manuscript should be published, and all data in the text should be available to the corresponding author.

## Funding

A grant project did not support this study.

## CRediT authorship contribution statement

**Yijia He:** Writing – original draft. **Shuanghu Dong:** Formal analysis, Methodology. **Lifen Ouyang:** Writing – original draft. **Jun Kang:** Data curation. **Liying Chen:** Methodology, Software. **Qingyun He:** Writing – review & editing.

## Declaration of competing interest

This study does not involve any conflict of interest.

## References

[1] Cecconi M., Evans L., Levy M., Rhodes A. (2018). Sepsis and septic shock. Lancet Lond. Engl..

[2] van der Poll T., Shankar-Hari M., Wiersinga W.J. (2021). The immunology of sepsis. Immunity..

[3] Rudd K.E. (2020). Global, regional, and national sepsis incidence and mortality, 1990-2017: analysis for the global burden of disease study. Lancet Lond. Engl..

[4] Brizuela V. (2020). Early evaluation of the ‘STOP SEPSIS!’ WHO global maternal sepsis awareness campaign implemented for healthcare providers in 46 low, middle and high-income countries. BMJ Open..

[5] Marshall J.C. (2014). Why have clinical trials in sepsis failed?. Trends Mol. Med..

[6] Whitehouse T. (2023). Landiolol and organ failure in patients with septic shock: the STRESS-L randomized clinical trial. JAMA..

[7] Downes K.J., Fitzgerald J.C., Weiss S.L. (2020). Utility of procalcitonin as a biomarker for sepsis in children. J. Clin. Microbiol..

[8] Tan M., Lu Y., Jiang H., Zhang L. (2019). The diagnostic accuracy of procalcitonin and C-reactive protein for sepsis: a systematic review and meta-analysis. J. Cell. Biochem..

[9] Hotchkiss R.S. (2016). Sepsis and septic shock. Nat. Rev. Dis. Primers..

[10] Jiao Y. (2021). Exosomal miR-30d-5p of neutrophils induces M1 macrophage polarization and primes macrophage pyroptosis in sepsis-related acute lung injury. Crit. Care Lond. Engl..

[11] Yao R.-Q. (2023). Single-cell transcriptome profiling of sepsis identifies HLA-DRlowS100Ahigh monocytes with immunosuppressive function. Mil. Med. Res..

[12] Liu Y., Zhang S., Liu K., Hu X., Gu X. (2024). Advances in drug discovery based on network pharmacology and omics technology. Curr. Pharmaceut. Anal..

[13] Chen J., Lin A., Luo P. (2024). Advancing pharmaceutical research: a comprehensive review of cutting-edge tools and technologies. Curr. Pharmaceut. Anal..

[14] Yan S. (2024). Exploring the immune-related molecular mechanisms underlying the comorbidity of temporal lobe epilepsy and major depressive disorder through integrated data set analysis. Curr. Mol. Pharmacol..

[15] Darden D.B. (2021). A novel single cell RNA-Seq analysis of non-myeloid circulating cells in late sepsis. Front. Immunol..

[16] Qiu X. (2021). Dynamic changes in human single-cell transcriptional signatures during fatal sepsis. J. Leukoc. Biol..

[17] Kwok A.J. (2023). Neutrophils and emergency granulopoiesis drive immune suppression and an extreme response endotype during sepsis. Nat. Immunol..

[18] Scicluna B.P. (2015). A molecular biomarker to diagnose community-acquired pneumonia on intensive care unit admission. Am. J. Respir. Crit. Care Med..

[19] Amir E.D. (2013). viSNE enables visualization of high dimensional single-cell data and reveals phenotypic heterogeneity of leukemia. Nat. Biotechnol..

[20] Wolock S.L., Lopez R., Klein A. M. Scrublet (2019). Computational identification of cell doublets in single-cell transcriptomic data. Cell Syst..

[21] Ashuach T., Reidenbach D.A., Gayoso A., Yosef N. (2022). PeakVI: a deep generative model for single-cell chromatin accessibility analysis. Cell Rep. Methods..

[22] Chu T., Wang Z., Pe’er D., Danko C.G. (2022). Cell type and gene expression deconvolution with BayesPrism enables Bayesian integrative analysis across bulk and single-cell RNA sequencing in oncology. Nat. Cancer.

[23] Kang M. (2024). Mapping single-cell developmental potential in health and disease with interpretable deep learning. BioRxiv Prepr. Serv. Biol..

[24] Zheng G.X.Y. (2017). Massively parallel digital transcriptional profiling of single cells. Nat. Commun..

[25] Cao J. (2019). The single-cell transcriptional landscape of mammalian organogenesis. Nature..

[26] Jin S., Plikus M.V., Nie Q. (2025). CellChat for systematic analysis of cell-cell communication from single-cell transcriptomics. Nat. Protoc..

[27] Xu S. (2024). Using clusterProfiler to characterize multiomics data. Nat. Protoc..

[28] Wu T. (2021). clusterProfiler 4.0: a universal enrichment tool for interpreting omics data. Innov. Camb. Mass..

[29] Fan C. (2024). irGSEA: the integration of single-cell rank-based gene set enrichment analysis. Briefings Bioinf..

[30] Hao Y. (2024). Dictionary learning for integrative, multimodal and scalable single-cell analysis. Nat. Biotechnol..

[31] Liu H. (2024). Mime: a flexible machine-learning framework to construct and visualize models for clinical characteristics prediction and feature selection. Comput. Struct. Biotechnol. J..

[32] Cao C., Yu M., Chai Y. (2019). Pathological alteration and therapeutic implications of sepsis-induced immune cell apoptosis. Cell Death Dis..

[33] Kim H.J. (2023). Inebilizumab reduces neuromyelitis optica spectrum disorder risk independent of FCGR3A polymorphism. Ann. Clin. Transl. Neurol..

[34] Vergadi E., Ieronymaki E., Lyroni K., Vaporidi K., Tsatsanis C. (1950). Akt signaling pathway in macrophage activation and M1/M2 polarization. J. Immunol. Baltim. Md..

[35] Luyendyk J.P. (1950). Genetic analysis of the role of the PI3K-Akt pathway in lipopolysaccharide-induced cytokine and tissue factor gene expression in monocytes/macrophages. J. Immunol. Baltim. Md.

[36] Peng Y. (2023). An immune-related gene signature predicts the 28-day mortality in patients with sepsis. Front. Immunol..

[37] He H. (2022). Identification of a novel sepsis prognosis model and analysis of possible drug application prospects: based on scRNA-seq and RNA-seq data. Front. Immunol..

[38] Mao M. (2020). MicroRNA-195 prevents hippocampal microglial/macrophage polarization towards the M1 phenotype induced by chronic brain hypoperfusion through regulating CX3CL1/CX3CR1 signaling. J. Neuroinflammation..

[39] Ni Y. (2022). CX3CL1/CX3CR1 interaction protects against lipotoxicity-induced nonalcoholic steatohepatitis by regulating macrophage migration and M1/M2 status. Metabolism..

[40] Lee A.J., Ashkar A.A. (2018). The dual nature of type I and type II interferons. Front. Immunol..

[41] Grundler Groterhorst K., Mannell H., Pircher J., Kraemer B.F. (2019). Platelet proteasome activity and metabolism is upregulated during bacterial sepsis. Int. J. Mol. Sci..

[42] Dela Cruz M.C.P., Paner J.R.O., Nevado J.B. (2023). Identification of potential prognosticators for sepsis through expression analysis of transcriptomic data from sepsis survivors and nonsurvivors. Acta Med. Philipp..

[43] Yu J. (2022). Maintenance of glutamine synthetase expression alleviates endotoxin-induced sepsis via alpha-ketoglutarate-mediated demethylation. FASEB J. Off. Publ. Fed. Am. Soc. Exp. Biol..

[44] Identification and experimental validation of diagnostic and prognostic genes CX3CR1, PID1 and PTGDS in sepsis and ARDS using bulk and single-cell transcriptomic analysis and machine learning - PubMed. https://pubmed.ncbi.nlm.nih.gov/39763654/.

